# The role and applications of extracellular vesicles in osteoporosis

**DOI:** 10.1038/s41413-023-00313-5

**Published:** 2024-01-23

**Authors:** Fei Fang, Jie Yang, Jiahe Wang, Tiantian Li, Erxiang Wang, Demao Zhang, Xiaoheng Liu, Chenchen Zhou

**Affiliations:** 1 Institute of Biomedical Engineering, West China School of Basic Medical Sciences & Forensic Medicine, Sichuan University, Chengdu, 610041 China; 2 State Key Laboratory of Oral Diseases, National Clinical Research Center for Oral Diseases, West China Hospital of Stomatology, Sichuan University, Chengdu, 610041 China

**Keywords:** Osteoporosis, Bone

## Abstract

Osteoporosis is a widely observed condition characterized by the systemic deterioration of bone mass and microarchitecture, which increases patient susceptibility to fragile fractures. The intricate mechanisms governing bone homeostasis are substantially impacted by extracellular vesicles (EVs), which play crucial roles in both pathological and physiological contexts. EVs derived from various sources exert distinct effects on osteoporosis. Specifically, EVs released by osteoblasts, endothelial cells, myocytes, and mesenchymal stem cells contribute to bone formation due to their unique cargo of proteins, miRNAs, and cytokines. Conversely, EVs secreted by osteoclasts and immune cells promote bone resorption and inhibit bone formation. Furthermore, the use of EVs as therapeutic modalities or biomaterials for diagnosing and managing osteoporosis is promising. Here, we review the current understanding of the impact of EVs on bone homeostasis, including the classification and biogenesis of EVs and the intricate regulatory mechanisms of EVs in osteoporosis. Furthermore, we present an overview of the latest research progress on diagnosing and treating osteoporosis by using EVs. Finally, we discuss the challenges and prospects of translational research on the use of EVs in osteoporosis.

## Introduction

Osteoporosis is a bone disorder characterized by reduced bone density and compromised bone microstructure that leads to increased bone fragility and subsequent fractures.^1^ According to the definition of the World Health Organization, osteoporosis can be diagnosed when the bone mineral density falls below 2.5 standard deviations from the peak bone value of healthy adults of the same sex and race.^2^ The current burden of osteoporotic fractures worldwide is substantial, and the costs are projected to increase dramatically annually.^3^ The pathogenesis of osteoporosis involves an imbalance between bone formation by osteoblasts and bone resorption by osteoclasts.^4^ Pharmacological interventions for osteoporosis mainly include calcium, vitamin D, the estrogen receptor modulator raloxifene, the RANKL receptor agonist denosumab, the parathyroid hormone analog teriparatide, and abaloparatide.^5,6^ Although drug intervention is effective, it may cause adverse reactions or drug resistance.^7^ Hence, the development of novel therapeutic approaches for treating osteoporosis is urgently needed.

Extracellular vesicles (EVs) are small membrane-bound structures released by cells that are commonly found in the extracellular matrix, various bodily fluids, or cell culture supernatants.^8^ Depending on their mechanism and size, EVs can be divided into three types: exosomes (30–150 nm), microvesicles (MVs, 100–1 000 nm) and apoptotic bodies (ABs, 1–5 μm).^9^ Exosomes are released through the fusion of multivesicular bodies (MVBs) generated by the endoplasmic reticulum and Golgi apparatus with the cell membrane. MVs are formed by inward protrusions and severing of the cell membrane. ABs are the membrane fragments of apoptotic cells formed by wrapped organelles or DNA. The main contents of EVs are proteins, DNA, mRNAs, miRNAs, and lipids.^10^ EVs play diverse roles in biological processes and contribute to the pathogenesis of various diseases, including cardiovascular diseases,^11,12^ cancer,^13^ and bone diseases.^14^ EVs have garnered significant interest in disease diagnosis and treatment in recent years; thus, they have attracted the attention of researchers and scholars alike.^15^

EVs derived from different sources, such as osteoblasts, osteoclasts, and mesenchymal stem cells (MSCs), can regulate the balance between bone formation and bone resorption, thereby affecting the occurrence and development of osteoporosis. EVs can also serve as drug carriers to enhance the targeting and bioavailability of drugs in bone tissue, providing a promising strategy for diagnosing and treating osteoporosis.^16^ First, we reviewed the biology of EVs and then summarized the functions of EVs derived from different sources in osteoporosis. Furthermore, we reviewed the application and engineering methods for using EVs to diagnose and treat osteoporosis to provide a reference for further examining the function and potential role of EVs in bone metabolism.

## The biology of EVs

### The biogenesis of EVs

#### Exosomes

**Fig. 1:**
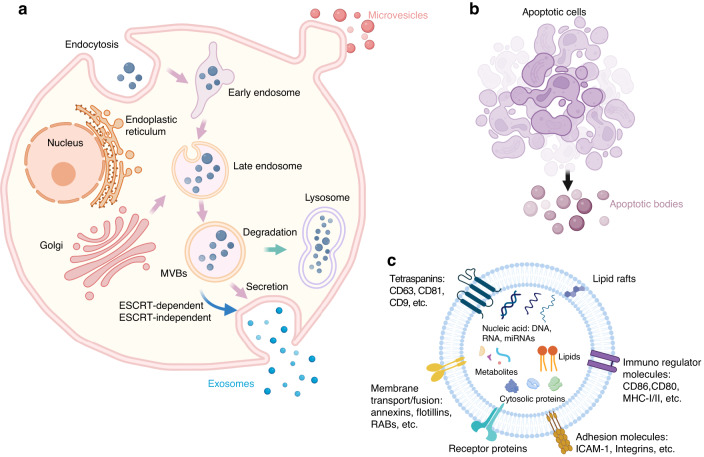
Biogenesis of EVs. **a** Microvesicles are produced by plasma membrane budding and blebbing. Extracellular components and cell surface proteins enter the cell through endocytosis and plasma membrane invagination to form early-sorting endosomes (ESEs), which can exchange materials with other organelles to form late-sorting endosomes (LSEs). LSEs further form intracellular MVBs and are degraded by fusion with autophagosomes or lysosomes or fuse with the plasma membrane to release their contents, including ILVs, as exosomes. **b** ABs are vesicles approximately 1–5 μm in diameter that are released from dying cells. **c** EVs contain many components, including lipids, DNA, RNA, and proteins

Exosomes are nanosized vesicles 30–150 nm in diameter that can be secreted by any cell.^17^ Exosomes originate from the development and maturation of MVBs, are transported to the plasma membrane space, fuse with the cell membrane and are expelled into the extracellular space (Fig. 1a).^18^ Recent studies have shown that exosomes can be directly released from the plasma membrane by budding into the extracellular space.^19,20^

Generally, exosome biogenesis mainly includes ESCRT-dependent and ESCRT-independent pathways.^21^ There are approximately 30 proteins involved in the ESCRT mechanism, four of which play essential roles: ESCRT-0, ESCRT-I, ESCRT-II, and ESCRT-III.^22^ These complexes function sequentially to regulate exosome biogenesis. The initial stages of intraluminal vesicle (ILV) formation and cargo packaging largely depend on the ESCRT-0 complex. The ESCRT-0 complex recruits the ESCRT-I complex to transmit the cargo by binding to the TSG-101 subunit.^23,24^ Next, ESCRT-I recruits ESCRT-II and, in conjunction with ESCRT-II, promotes invagination of the endosomal membrane.^25^ Ultimately, ESCRT-III is recruited by ESCRT-II, resulting in the dissociation of the membrane and the facilitation of ILV formation.^22,26^

Moreover, exosome biogenesis can occur independently of the ESCRT pathway.^27^ For instance, proteins belonging to the tetraspanin family mediate cargo loading and exosome secretion by clustering together and sequestering other proteins, thereby forming tetraspanin-rich microdomains.^28^ Importantly, CD9, CD53, CD63, CD81, and CD82 are essential regulators of ESCRT-independent formation of MVBs.^28,29^

#### Microvesicles (MVs)

MVs are a subtype of EV with diameters ranging from 100 to 1 000 nm that are formed by budding from the plasma membrane, but the mechanism of their biogenesis is not well understood.^30^ Numerous studies have suggested that, similar to exosome biogenesis, ESCRT-dependent mechanisms might be involved in the biogenesis of MVs.^31,32^ Furthermore, acid sphingomyelinase has been implicated in MV biogenesis as another regulator of ceramide.^33^ Increasing evidence has demonstrated that small GTPases, including those of the Rho family and ARFs, drive the budding of MVs from the plasma membrane.^34–37^

#### Apoptotic bodies (ABs)

ABs are vesicles approximately 1–5 μm in diameter that are released from dying cells; these vesicles differ in size, structure, and composition from MVs and exosomes (Fig. 1b).^38^ Apoptosome components include degraded proteins, DNA fragments, micronuclei, and even complete organelles.^39^ Previously, the contents of ABs were believed to be mostly useless waste that was phagocytosed by surrounding macrophages and degraded in lysosomes.^40^ ABs can be used as intercellular communication factors to directly regulate the activities of target cells.^41–43^

### EV internalization

After being released from source cells, EVs can adhere to the extracellular matrix and neighboring cells or be transferred to distant organs via blood, lymph, and other humoral pathways.^11^ After interacting with cells, EVs can mediate intercellular signaling through two primary modes: (1) they can transfer information to recipient cells through direct contact with surface ligands; or (2) they can transfer their contents (proteins, nucleic acids, DNA, microRNAs) to target cells.^44^

**Fig. 2:**
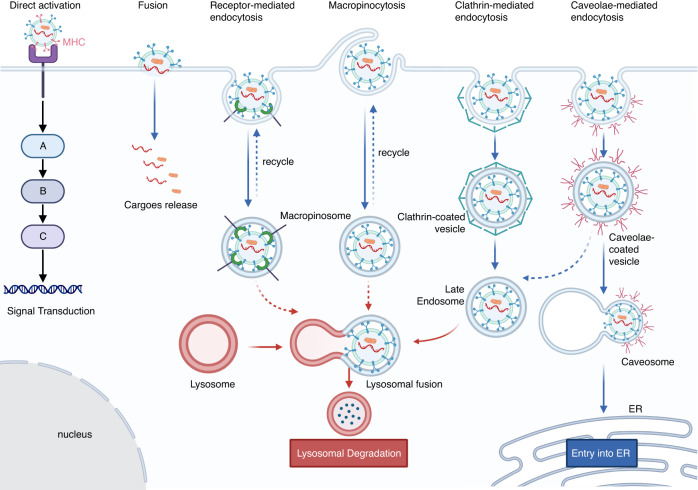
The pathway and fate of EVs after internalization. MHC molecules carried by EVs can directly activate signal transduction in acceptor cells (the downstream signaling molecules A, B and C have no specific reference). EVs can enter cells through membrane fusion, receptor-mediated endocytosis, macropinocytosis, clathrin-mediated endocytosis, and caveolae-mediated endocytosis. After entering the cell, most EVs fuse with lysosomes and are degraded

The first approach involves the interaction of EVs with target cells via membrane-bound ligand–receptor pairs, thereby initiating intracellular signaling pathways (Fig. 2). Typically, EVs secreted by immune cells, such as B cells and dendritic cells, carry major histocompatibility complex molecules on their surface, which can regulate the immune response of T cells.^45,46^ In addition, increasing evidence has confirmed that the immune escape function of tumor cells is caused by the binding of tumor cell-derived EVs carrying PD-L1 to the surface receptor PD1 on T cells.^47,48^

The second way involves the internalization of EVs after fusion with the acceptor cell membrane and the release of EV contents into the acceptor cell (Fig. 2). Numerous studies have shown that some receptor‒ligand pairs, such as integrins, heparan sulfate proteoglycans, tetraspanins, and tetherin, are involved in EV adhesion to recipient cells.^49–52^ However, due to the molecular complexity of the EV surface, identifying the exact receptors that mediate EV adhesion to recipient cells is difficult. Of course, multiple receptor–ligand pairs are likely to be cooperatively involved in this process. In addition, studies have shown that EV internalization may involve other pathways, including macropinocytosis, phagocytosis, clathrin-mediated endocytosis, and caveolin-dependent endocytosis.^11^ Although this approach has been extensively studied and characterized, there is still no consensus.

The fate of EVs after being internalized by cells is an essential factor that affects their functions. Generally, EVs follow rules similar to those of other substances after internalization. After fusion with early endosomes, they can be transferred to the plasma membrane and recycled or transferred to lysosomes for degradation (Fig. 2).^53^ There is evidence that fluorescently labeled EVs can accumulate in lysosomes after being internalized.^54,55^ Given the biological function of lysosomes, we believe that the cargoes of EVs that enter lysosomes will be degraded and unable to perform their functions. However, considerable evidence indicates that EV internalization can significantly affect the function of recipient cells, suggesting that cargo-loaded EVs can somehow escape lysosomal engulfment.

### EV cargo

The cargo composition and sorting mechanism of EVs have been relatively fully characterized.^56^ Here, we provide a brief review of cargo sorting for EVs. EVs contain various substances, such as proteins, lipids, RNA, and DNA, and their composition can be the same or different from that of the source cell.

#### Proteins

The ESCRT mechanism plays a key role in sorting proteins in EVs. As mentioned previously, the ubiquitination-binding domain of ESCRT can bind ubiquitinated proteins and is necessary for protein sorting. The ESCRT complex prevents the degradation of ubiquitinated cargo and deforms the membrane to sort the cargo into ILVs.^57^ In addition, due to the differences in the composition and function of the four subcomplexes of ESCRT, the proteins sorted at different stages of EV formation also differ. There are also protein sorting pathways that are not dependent on the ubiquitination pathway. For example, SUMOylation, ISGylation, phosphorylation, and oxidation can regulate the interaction between exosome loading and various posttranslational modifications (PTMs, signals for cargo transport to MVBs).^58^

#### RNA

The RNAs contained in EVs significantly differ at the cellular level, indicating a unique mechanism for RNA sorting in EVs.^59^ RNAs in EVs include miRNAs, mRNAs, tRNAs, and small nucleolar RNAs. RNA-binding proteins (RBPs) containing sequence-specific RNA-binding domains act as adapters between the RNA cargo and the EV biogenesis machinery, which is a key mechanism that regulates RNA cargo sorting.^60^ Many RBPs, such as hnRNPA2B1,^61^ hnRNPK,^62^ YBX1,^63^ major vault protein (MVP),^64^ and Argonaute-2,^65^ have been suggested to be involved in RNA sorting in different cell models.

#### DNA

Although the mechanism of DNA sorting into EVs has not been fully characterized, it is clear that EVs also contain DNA and DNA fragments. In most cases, DNA is adsorbed on the surface of EVs, but studies have confirmed that DNA is present within EVs.^66,67^ Interestingly, DNA is more likely to be present in large EVs than in small EVs.^68^ Studies have shown that the reason for the DNA in tumor cell-derived EVs is that cytoplasmic micronuclei interact with tetraspanins to sort DNA into EVs.^69^ In addition, after mitochondria interact with MVBs, DNA can also be transferred to EVs and released into the extracellular space.^70^

### EV isolation

Different EV isolation methods significantly affect the purity and yield of EVs. The acquisition of pure EVs is limited by the challenges associated with their nanoscale size and by the contamination of other factors that are isolated with EVs, such as cell debris, proteins, and other substances.^71^

**Fig. 3:**
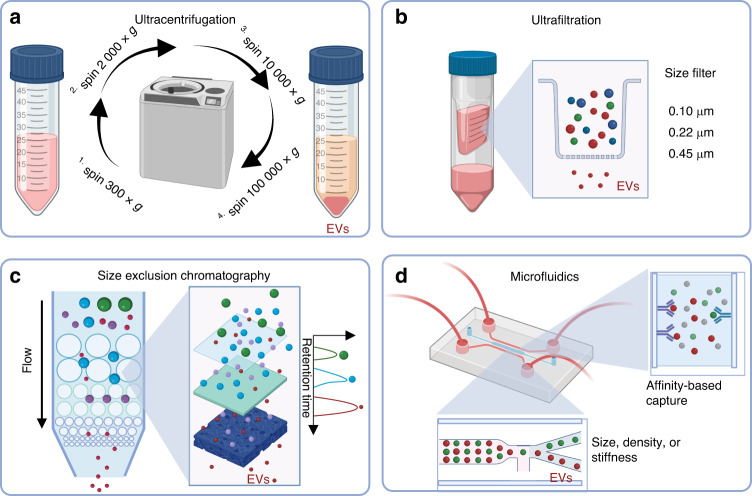
The main methods of EV isolation. **a** Ultracentrifugation: EVs are obtained by a programmed gradient centrifugation method. **b** Ultrafiltration: EVs with different particle sizes are separated by a filter membrane (including 0.10 μm, 0.22 μm, and 0.45 μm) with a specific pore size. **c** Size exclusion chromatography, SEC: Pure EVs are eluted and separated according to the retention times of EVs and other components in the column. **d** Microfluidic technology: According to the specific affinity adsorption, size or density characteristics of EVs, narrow microchannels can be designed to capture them

Ultracentrifugation is the mainstream method for separating EVs and is simple and easy to perform without the support of commercial kits (Fig. 3a). This method requires the application of a centrifugal force of 12 000 × *g* to the sample for 2 h at 4 °C. To further improve the purity of the EVs, density gradient centrifugation can be used.^72^ Currently, iodixanol or sucrose solutions are the most commonly used separation media for dispersing EVs in specific density regions.^73^ Centrifugation has been used to obtain high-purity EVs, but this method is labor intensive and unsuitable for high-throughput applications.

Ultrafiltration is a widely used approach for separating EVs based on size (Fig. 3b). EVs can be obtained by filtering impurities through a simple membrane filter with a specific size exclusion limit (e.g., 0.10, 0.22, or 0.45 μm pore size). The problem with this separation method is that it cannot remove contaminants (such as proteins) that are smaller than the filter pore size.^74^ Typically, ultrafiltration and ultracentrifugation can be combined to obtain high-purity EVs.

Size exclusion chromatography (SEC) has been widely used to separate EVs from various samples, including cells, blood, and body fluids (Fig. 3c).^75,76^ Larger molecules cannot enter the column and flow out quickly through the pores, while smaller molecules elute slowly through the pores of the stationary phase.^77^ EVs are bulky molecules that can be rapidly eliminated through the pores without being retained in the column. SEC has multiple advantages, such as maintaining the structural integrity of EVs, ensuring high purity, and meeting low infrastructure requirements.^77–79^ A study revealed that SEC yielded more pure exosomes than ultracentrifugation.^78,80^ The main disadvantage of SEC in EV enrichment is the inability to distinguish other components similar in size to EVs, such as LDL (25 nm), VLDL (30–80 nm), and chylomicrons (75–1 200 nm).^81–83^ However, recent studies have developed new methods based on SEC to separate EVs and LDL.^84,85^

Several microfluidic platforms have been used to rapidly and efficiently isolate EVs derived from biological fluids with higher recovery and purity than ultracentrifugation.^86^ Microfluidics offers distinct advantages, such as low sample consumption, precise fluidic control, high resolution and throughput, and short processing times (Fig. 3d).^87^ Microfluidic technologies for EV isolation can be classified into two categories: EV separation based on physical properties (size, density, or stiffness) and affinity-based capture. Microfluidics-based EV isolation is often integrated with molecular detection techniques for disease diagnosis.^88^ EV separation by microfluidics has extensive application prospects in disease diagnosis.

In addition, discussions on EV isolation, such as isolation by precipitation, affinity capture, and commercial kits, have been well summarized.^74^ With an in-depth understanding of the physical characteristics and biomarkers of EVs, more efficient ways to obtain high-purity and highly active EVs will be developed.

## The potential role of EVs derived from different sources in osteoporosis

**Fig. 4:**
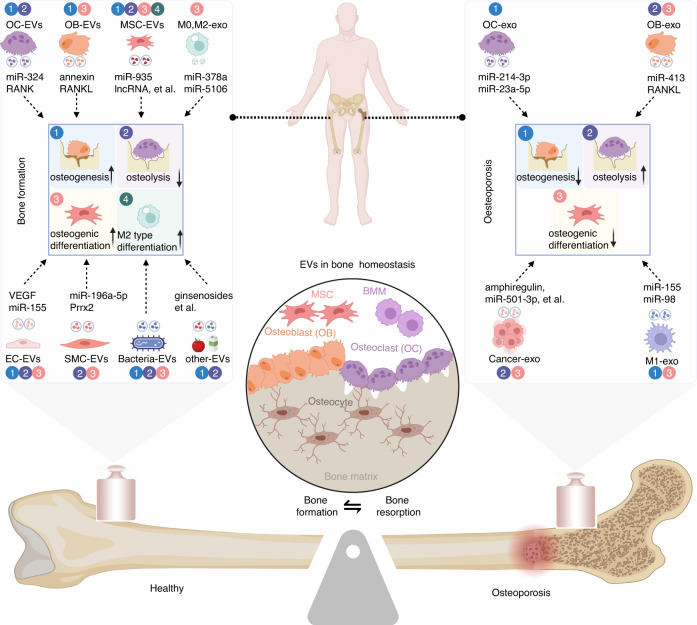
Roles of EVs derived from different sources in bone homeostasis. EVs derived from osteoblasts, osteoclasts, MSCs, endothelial cells, and muscle cells can increase bone formation and inhibit bone resorption by promoting the activity of osteoblasts, inhibiting the activity of osteoclasts, and promoting the differentiation of MSCs. Exosomes derived from osteocytes, osteoclasts, tumor cells, and M1 macrophages inhibit bone formation by inhibiting osteoblast activity, promoting osteoclast activity, and inhibiting the osteogenic differentiation of MSCs. The numbers in the circles next to each type of EV correspond to the numbers of key regulatory factors involved in the bone formation or resorption process in the figure

EVs derived from tissues, cells, or body fluids, as well as those that originate from plants and bacteria, have been shown to regulate the delicate balance of bone homeostasis (Fig. 4). Typically, osteoblasts, osteoclasts, and MSCs are the primary target cells for these EVs. This section focuses on the role of EVs derived from diverse sources in osteoporosis. A summary is presented in Table 1 to provide a comprehensive overview.

**Table 1:** The EVs from different sources and their functions

Donor cell	EVs type	Stimulation/Condition	Cargo	Acceptor cell	Function	Ref.
osteoclasts	Small EVs	-	miR-324	MSCs	Induced osteogenic differentiation of MSCs	^89^
	EVs or ABs	-	RANK	osteoblastic	Binds osteoblastic RANKL promotes bone formation	^90,91^
	exosome	-	miR-23a-5p	osteoblastic	Inhibit osteogenic differentiation by Runx2	^92^
	exosome	-	miR-214-3p	osteoblastic	Inhibit bone formation	^93^
osteoblasts	EVs	-	annexin	BMSCs	Induce mineralization in MSC cultures	^96^
	exosome	TiO_2_ NPs	-	HMSCs	Decrease HMSC osteogenic differentiation	^97^
	EVs	osteoporosis		BMSCs	Inhibit BMSCs osteogenic differentiation	^98^
	exosome	-	miR-503-3p	osteoclasts	Inhibit osteoclast differentiation by downregulating the expression of Hpse	^99^
	microvesicle	-	RANKL	osteoclasts	Facilitate osteoclast formation through RANKL–RANK signaling	^100^
	exosome	-	Circ_0008542	osteoclasts	Improve osteoclast differentiation by increased expression of m6A methylation	^101^
	small vesicles	-	miR-143	osteoblastic	Suppress osteoblast differentiation by inhibits Runx2	^102^
HBMSCs	exosome	-	miR-935	osteoblastic	Enhance osteoblast proliferation and differentiation in osteoporotic rats	^105^
	exosome	-	lncRNA	osteoblastic	Alleviates osteoporosis through microRNA-34c/SATB2 axis	^107^
BMSCs	exosome	-	lncRNA-lncTUG1	osteoblastic	Promote bone formation via miR-22-5p/Anxa8 axis	^106^
	EVs	-	miR-22-3p	BMSCs	Enhance osteogenic differentiation by inactivate the MYC/PI3K/AKT pathway	^109^
	exosome	-	-	BMSCs	Enhance the osteogenic capacity of older BMSCs and promote bone formation	^110^
	exosome	-	-	osteoblast	Promote bone regeneration	^112^
	exosome	-	-	BMSC/macrophage	Induce osteogenic differentiation of BMSCs and inhibits M1-type polarization of macrophages	^117^
MSCs	exosome	-	-	osteoclasts	Alleviate diabetic osteoporosis by suppressing NLRP3 inflammasome activation	^108^
	exosome	aged rats	miRNA-128-3p	MSCs	Promote bone fracture healing	^111^
	exosome	hypoxic	miR-126	HUVECs	Promote angiogenesis by enhance bone healing	^115^
	exosome	-	-	chondrocytes macrophage	Promote chondrocytes proliferation and increase M2 type macrophage population	^116^
	exosome	miR-21 transfected	miR-21	-	Alleviate spinal osteoporosis	^118^
hucMSCs	exosome	hydrogel		MC-3T3	Repair bone defects in rats	^113^
	exosome	hydrogel	-	osteoblast	Accelerate fracture healing via the promotion of angiogenesis	^114^
macrophage	EVs	-	miR-378a/miR-155	MSC	M0, M2 EVs promoted regeneration and M1 EVs inhibited bone repair	^120^
	exosome	M1 type	miR-98	MC3T3	Exacerbates bone loss by downregulating the DUSP1-JNK pathway	^121^
	exosome	M1 type	miR-21a-5p	BMSCs	Promote osteogenesis of BMSCs	^122^
	exosome	M2 type	miR-5106	BMSCs	Promote osteogenic differentiation of BMSCs and accelerate fracture healing	^123^
VECs	exosome	VEGF transfected	VEGF	BMSCs	Promoted osteoblast differentiation and suppressed adipogenic differentiation	^128^
	exosome	-	-	osteoblast	Reverses osteoporosis by inhibiting osteoblast ferroptosis	^129^
	exosome	-	Lnc NEAT1	macrophage	Inducing M2 polarization of macrophages through DDX3X/NLRP3 regulatory axis	^131^
	exosome	-	miR-155	osteoclast	Inhibit osteoclast activity and inhibit osteoporosis in mouse model	^132^
EPCs	EVs	-	-	osteoblast	Prevent steroid-induced osteoporosis by suppressing the ferroptotic pathway	^130^
muscle cells	EVs	-	-	BMSC/osteoclast	Promote osteogenic differentiation of BMSC and inhibit osteoclast formation	^136^
	EVs	-	-	osteoclast	Suppress osteoclast formation and mitochondrial energy metabolism	^137^
	EVs	-	miR-196a-5p	osteoclast	Inhibit osteoclast formation	^138^
	exosome	-	Prrx2	BMSCs	Promote osteogenic differentiation of BMSC by the MIR22HG-YAP pathway	^139^
	EVs	TNF-α	-	osteoclast	Blunts both the osteoclast formation suppression and the osteoblastic differentiation promotion	^140^
	EVs	aging	miR-12a	BMSCs	Decrease Sirt1 expression and increase BMSC senescence	^141^
multiple myeloma	exosome	-	-	osteoblast/osteoclast	Enhance osteoclast activity and block osteoblast differentiation	^143^
	exosome	-	amphiregulin	BMSCs	Inhibit the osteogenic of BMSC	^144^
	exosome	-	lncRUNX2-AS1	MSCs	Inhibit the osteogenesis of MSCs	^145^
osteosarcoma	exosome	-	miR-501-3p	BMDM	Promote osteoclast differentiation via PTEN/PI3K/Akt signaling pathway	^146^
breast cancer	exosome	-	miR-20a-5p	bone marrow macrophage	Promote osteoclasts proliferation and differentiation by targeting SRCIN1	^147^
NSCLC	exosome	-	miR-17-5p	osteoclast	Promote osteoclastogenesis through the PI3K/Akt pathway via targeting PTEN	^148^
pancreatic cancer	exosome	-	miR-125a-5p	osteoclast	Induce osteoclast differentiation	^149^
human umbilical cord blood	EVs	-	miR-3960	osteoblast/osteoclast	Promote osteoblast differentiation and inhibit osteoclast differentiation	^151^
urine-derived stem cells	EVs	-	miR-26a-5p	osteoblast/osteoclast	Enhance the activity of osteoblasts and inhibit the activity of osteoclasts	^152^
	EVs	-	CTHRC1, OPG	osteoblast/osteoclast	Promote osteogenesis and inhibit osteoclastogenesis	^153^
Amniotic fluid stem cell	EVs	-	-	osteoblast	Alleviate dexamethasone-induced inhibition of osteoblast differentiation	^154^
Escherichia coli	EVs	siRNA loading	-	BMSCs	Induce osteogenic differentiation of BMSCs by regulating the WNT signaling	^156^
Akkermansia muciniphila	EVs	-	-	osteoblast/osteoclast	Promote osteogenic differentiation of osteoblasts and inhibit the action of osteoclasts	^157^
milk	exosome	-	-	osteoclast	Inhibit osteoclast differentiation	^150^
yam	exosome	-	-	osteoblast	Stimulate the proliferation, differentiation, and mineralization of osteoblasts	^164^
ginseng	exosome	-	ginsenosides	osteoclast	Inhibit osteoclast differentiation	^165^
plum	exosome	-	-	osteoblast/osteoclast	Improve osteoblast differentiation and inhibit osteoclast activation	^166^
apple	Nanovesicle	-	-	osteoblast	Promote osteoblastogenesis through BMP2/Smad1 pathways	^167^

### Osteoclast-derived EVs

Osteoclasts are one of the key cell types involved in bone homeostasis, and their main function is to resorb the bone matrix. Osteoclast-derived EVs (OC-EVs) play an important role in bone homeostasis. Studies have confirmed that OC-EVs enriched with miR-324 can significantly promote the osteogenic differentiation of MSCs by targeting ARHGAP1, a negative regulator of osteogenic differentiation.^89^ Interestingly, the roles of OC-EVs in osteoblast differentiation are quite different. One study revealed that osteoclast-derived small EVs containing RANK promoted osteoblast differentiation through RANKL reverse signaling.^90^ In addition, osteoclast-derived apoptotic bodies can induce osteogenic differentiation in MSCs and promote osteoblastic differentiation through RANKL reverse signaling.^91^ However, osteoclast-derived exosomes inhibited osteoblastic bone formation by delivering miR-23a-5p^92^ and miR-214-3p.^93,94^ Zhang et al. reported that an increase in osteoclast miR-214-3p was associated with increased serum exosome miR-214-3p levels and decreased bone formation in older women with fractures and ovariectomized (OVX) mice.^93^ Furthermore, osteoclast-derived exosomal miR-214-3p was transferred into osteoblasts to suppress osteoblast activity in vitro and reduce bone formation in vivo.^93^ An investigation of the size distribution of OC-EVs in the literature revealed that OC-EVs with a particle size less than 100 nm inhibited the osteogenic differentiation of osteoblasts. In contrast, OC-EVs with a particle size exceeding 100 nm exhibited enhanced potential to induce osteogenic differentiation in osteoblasts.

### Osteoblast-derived EVs

Osteoblasts are the primary functional cells involved in bone formation and are mainly responsible for bone matrix secretion, synthesis, and mineralization. Studies have shown that EVs are essential for paracrine signaling transmission by osteoblasts.^95^ Osteoblast-derived EVs can promote the osteogenic differentiation of bone marrow mesenchymal stem cells (BMSCs) through the attachment of EV-associated annexin to sites of mineral accumulation and nucleation.^96^ In contrast, EVs secreted by osteoblasts within the pathological microenvironment inhibited the osteogenic differentiation of MSCs.^97,98^ Interestingly, there is a lack of consensus regarding the regulatory effects of osteoblast-derived EVs on osteoclast differentiation. Li et al. demonstrated that osteoblast-derived exosomes enriched with miR-503-3p suppressed osteoclast differentiation by downregulating heparanase gene expression.^99^ However, Fu et al. reported that osteoblast-derived MVs contained the RANKL protein, which can promote osteoclast differentiation.^100^ In addition, another study confirmed that Circ_0008542 enrichment in osteoblast-derived exosomes promoted osteoclast-induced bone resorption by acting as a miR-11-185p sponge to upregulate RANK gene expression.^101^ A recent study provides a plausible explanation for this confusion. Ishii et al. reported that mature osteoblast-derived EVs can be divided into two subsets.^102^ Although these two subsets expressed EV surface markers, their particle sizes differed by approximately 200 nm and 400 nm.^102^ Among them, only small osteoblast vesicles with a particle size of approximately 400 nm were rich in miR-143-3p, which inhibited osteoblast differentiation and stimulated osteoclast formation by targeting Cbfb mRNA.^102^ This intriguing phenomenon suggests that EVs originating from the same cell but varying in size may exhibit distinct biological functions.

### EVs derived from MSCs

MSCs are mesoderm-derived adult stem cells that have a remarkable capacity for self-renewal and multilineage differentiation, enabling them to give rise to diverse mesenchymal tissues. Numerous investigations have used direct local injection of MSCs as a treatment for osteoporosis. These cells can self-renew, migrate to the injury site, differentiate into osteoblasts, and regulate the immune system at the injury site, rendering them valuable factors for bone tissue regeneration.^103^ Currently, the application paradigm of MSCs has shifted from a differentiation- and replacement-based approach to one centered around the use of secreted and paracrine effectors.^104^

EVs derived from MSCs exhibit promising potential for the treatment of osteoporosis; these cells can promote osteoblast activity, inhibit osteoclast differentiation, promote osteogenic differentiation in BMSCs, and regulate immune functions. BMSC-exos enriched in miR-935 inhibited STAT1 expression in osteoblasts, promoted osteoblast mineralization and nodule formation and enhanced ALP activity.^105^ BMSC-derived exosomal lncTUG1 enhanced osteoblast activity and promoted fracture recovery in vivo through the miR-22-5p/Anxa8 axis.^106^ MALAT1 in BMSC-derived exosomes enhanced osteoblast activity in osteoporotic mice by mediating the miR-34c/SATB2 axis.^107^ However, there have been few studies on the regulatory effect of MSC-derived exosomes on osteoclast differentiation. Exosomes derived from adipose MSCs alleviated bone loss in diabetic rats with osteoporosis by inhibiting NLRP3 inflammasome activation and the secretion of IL-1β and IL-18 by osteoclasts.^108^

There are many studies on the regulatory effect of MSC-derived exosomes on the osteogenic differentiation of BMSCs. MSC-EVs affect the osteogenic differentiation of MSCs through multiple pathways. For example, BMSC-derived EVs were enriched in miR-22-3p, which promoted BMSC osteogenic differentiation through fat mass and obesity-associated protein inhibition by inhibiting the MYC/PI3K/AKT pathway.^109^ Exosomes secreted by young MSCs promoted bone regeneration in aged rats by enhancing the proliferation and osteogenic capacity of BMSCs.^110^ Conversely, exosomes from aged rat MSCs were enriched in miRNA-128-3p and suppressed osteogenesis by targeting Smad5.^111^ Furthermore, the use of scaffold materials to encapsulate MSC-EVs has shown promising outcomes in bone regeneration, demonstrating their remarkable therapeutic efficacy.^112,113^

MSC-EVs can also regulate bone angiogenesis to promote bone formation. MSC-derived EVs promoted the proliferation and migration of HUVECs, increased tube formation and upregulated angiogenesis-related genes, such as VEGF and HIF-1α.^114^ A recent study demonstrated that hypoxia-preconditioned MSCs activated HIF-1α to produce miR-126-enriched exosomes.^115^ These EVs can be transferred into HUVECs to target SPRED and activate Ras/ERK signaling; promote proliferation, migration and angiogenesis in HUVECs; and promote fracture healing.^115^ Furthermore, MSC-EVs can promote osteogenesis by balancing the bone immune microenvironment. MSC-derived exosomes increase M2 macrophage infiltration and reduce the population of M1 macrophages and the expression of proinflammatory cytokines to promote osteogenesis.^116–118^

### EVs derived from macrophages

Bone-resident macrophages can regulate bone metabolism by secreting many cytokines and exosomes to communicate with other osteocytes.^119^ Previous studies have shown that macrophage polarization plays an important role in regulating the differentiation of MSCs and the activity of osteoblasts.^120^ miRNA sequencing studies have shown that the miRNAs of M0 and M2 macrophages are similar but significantly different from those of M1 macrophages.^120^ Studies have shown that M1 macrophage-derived EVs (M1-EVs) are rich in miRNA-155, which can decrease the expression of BMP2, BMP9, and RUNX2 to inhibit the osteogenic differentiation of MSCs.^120^ Ma et al. also reported that M1-EVs could aggravate postmenopausal osteoporotic bone loss through the microRNA-98/DUSP1/JNK axis.^121^ In contrast, M2 macrophage-derived EVs (M2-EVs) can promote the osteogenic differentiation of MSCs. One study revealed that miR-378a,^120^ miR-21a-5p.^122^ or miR-5106.^123^ in M2-EVs may be key factors for osteogenic differentiation. M2-EVs carrying miR-5106 targeted the salt-inducible kinase 2 and 3 (SIK2 and SIK3) genes to promote osteogenic differentiation in BMSCs and accelerate femoral fracture healing in mice.^123^ These studies suggest that the distinct states of macrophage-derived EVs play different roles in bone homeostasis. Therefore, EVs secreted by macrophages that induce an anti-inflammatory phenotype may be candidates for the treatment of osteoporosis. There have been reports on this strategy, such as inducing macrophages into an osteoprotective phenotype through mechanical force.^124^ or titanium dioxide nanotubes.^125^ and using EVs secreted by these cells to treat osteoporosis.

### EVs derived from endothelial cells

The cardiovascular system significantly contributes to the functionality of the skeletal system.^126^ As an essential component of blood vessels, ECs are located in the inner layer of blood vessels and often internalize and secrete substances.^127^ Studies have shown that EVs secreted by ECs (EC-EVs) can improve the activity and functions of osteocytes induced by steroids.^128,129^ Mechanistically, EC-EVs play an antiosteoporotic role by inhibiting osteocyte ferroptosis.^129^ A similar phenomenon occurs when EVs are derived from endothelial progenitor cells (EPCs), which can reverse osteoporosis induced by large doses.^130^ However, there have been few reports of active agents within ECs-EVs that can treat osteoporosis. Previous studies have confirmed that LNCRNAs.^131^ and miRNAs.^132^ in ECs-EVs may be involved in osteoporosis. Su et al. reported that miR-155 in EC-EVs could ameliorate osteoporosis in vitro and in vivo.^132^ Interestingly, the authors compared the effects of exogenous EV injection on the distribution of ECs, BMSCs, and bone cells, and found that only ECs-EVs were enriched in bone tissue.^132^ The author speculated that the protein (PZP) expressed in these ECs-EVs may be the leading cause of this phenomenon.^132^ Generally, the evidence suggests that EC-EVs promote osteoma to inhibit osteoporosis.

### EVs derived from muscle cells

Skeletal muscles and bones are the two main components of the musculoskeletal system. The direct mechanical interaction between muscle and bone has been well characterized over the past few decades. Research in the past decade has shown that the interaction between muscles and bone exceeds mechanical actions.^133^ For example, bone repair in a mouse model of open tibial fractures was notably amplified in the fracture region encompassed by a muscle flap.^134^ Conversely, if the muscle is severely damaged, fracture healing will be delayed.^134^ These findings suggest that muscle and bone communication occur through the secretion of biochemical factors. EV-mediated signaling in muscle and bone is an exciting emerging field, but the underlying mechanisms remain to be explored.^135^ Studies have confirmed that EVs derived from healthy skeletal muscle cells can promote the osteogenic differentiation of BMSCs and inhibit the formation of monocytic osteoclasts.^136–138^ However, there have been few reports on the mechanism by which myocyte-derived EVs regulate osteoporosis. He et al. confirmed that the high expression of Prrx2 in C2C12 cell-derived EVs directly combined with the MIR22HG promoter and promoted its transcription and expression, after which the sponge miR-128 enhanced the expression and nuclear translocation of YAP, thereby promoting osteogenic differentiation in BMSCs.^139^ It has also been reported that myocyte-derived EVs stimulated by atrophic muscle,^136^ inflammation,^140^ or oxidative stress.^141^ can induce osteoblast senescence and aggravate osteoporosis. EVs derived from muscle cells can regulate bone homeostasis. However, the molecular mechanism of EV activation, transport, and regulation of bone homeostasis remain to be further explored.

### EVs derived from tumor cells

The relationship between tumors and bone diseases has received increased attention. Osteolysis is an important feature of in situ bone tissue tumors (such as multiple myeloma and osteosarcoma) and bone metastatic tumors.^142^ To date, only a few studies have reported the crosstalk of EVs between tumors and bone diseases. For examine, multiple myeloma has been well studied, and 60% of patients have osteolytic lesions.^143^ Menu et al. reported that EVs derived from multiple myeloma cells not only enhanced the activity of osteoclasts but also inhibited the activity of osteoblasts by reducing the expression of Runx2, Osterix and collagen-1A in osteoblasts by mediating the transfer of DKK-1.^143^ Moreover, other evidence indicates that multiple myeloma-derived EV-rich amphiregulin (AREG).^144^ and lncRUNX2-AS1.^145^ may be critical factors that promote osteoclast activity or inhibit osteoblast activity. In addition, EVs derived from other tumors, such as osteosarcoma,^146^ breast cancer,^147^ non-small cell lung cancer,^148^ and pancreatic cancer,^149^ have been confirmed to promote osteoclast differentiation and aggravate bone calcium flow. According to the existing reports, a consensus can be reached that EVs derived from tumor cells can promote bone calcium loss and induce osteoporosis or fractures.

### EVs derived from biological fluids

EVs are widely present in all biological fluids, such as blood, urine, milk, saliva, and amniotic fluid.^150^ Studies have shown that EVs found in biological fluids play important roles in regulating bone homeostasis. Blood-derived EVs can serve as diagnostic markers for osteoporosis, which will be extensively discussed in Section 4.1. Anecdotal evidence suggests that EVs derived from human umbilical cord blood can mitigate bone loss in aged osteoporotic mice.^151^ Urine-derived EVs have received much attention because urine-derived stem cells have good proliferative activity and multilineage differentiation potential. Research has revealed that urinary stem cell-derived EVs are enriched in miR-26a-5p, which promotes osteoblast differentiation and inhibits osteoclast activity in osteoblast precursor cells.^152^ Zhang et al. also reported that urine-derived stem cell-derived EVs protect against osteoporosis, and CTHRC1 and OPG, which are enriched in EVs, are critical components that promote osteogenesis and inhibit osteoclasts.^153^ Furthermore, studies have reported that EVs derived from bovine milk.^150^ and amniotic fluid.^154^ also exhibit antiosteoporotic properties.

### EVs derived from bacteria

The relationship between bacterial extracellular vesicles (BEVs) and osteoporosis requires further understanding the gut-bone axis theory, and increasing evidence supports the important role of the gut microbiota in bone homeostasis and the pathogenesis of osteoporosis.^155^ The gut microbiota, especially probiotics (such as *LGG*,^156^ *Akkermansia muciniphila (AKK)*,^157^ *Lactobacillus reuteri*,^158^ *Lactobacillus paracasei*.^159^ and *Bifidobacterium longum*.^160^), has become an important therapeutic agent for osteoporosis.

BEVs are vesicles with a phospholipid bilayer that are released by most bacteria. Various molecules, including nucleic acids, proteins, lipids, and metabolites, are enriched in BEVs and mediate communication between bacteria and hosts, thus playing an important role in the regulation of physiological and pathological processes.^161^ For example, treating OVX mice with *AKK*-derived BEVs can promote osteogenic differentiation in osteoblasts and inhibit the action of osteoclasts.^157^ Recently, Su et al. reported the use of engineered probiotic EVs for the treatment of osteoporosis, and these engineered EVs (BEV-CSs) could be internalized by bone marrow MSCs to promote their osteogenic differentiation and ultimately ameliorate osteoporosis.^156^

The nanostructure, cell-free system, good biocompatibility and low toxicity of BEVs have emerged as promising platforms for biomedical applications. In addition, the advantages of rapid proliferation and well-established high-density bacterial culture enable large-scale production of BEVs.^162,163^

### EVs derived from plants

EVs secreted by plants contain mRNAs, proteins, miRNAs, and bioactive lipids with unique and diverse pharmacological mechanisms that can exert multiple effect, such as antioxidant, anti-inflammatory, and antiosteoporotic effects. Studies have reported that EVs isolated from yams,^164^ ginseng,^165^ plums,^166^ and apples.^167^ have antiosteoporotic effects. In a recent study, yam-derived EVs (YNVs) were successfully extracted and characterized by ultracentrifugation.^164^ YNVs stimulated the proliferation, differentiation, and mineralization of osteoblasts; increased the expression of bone differentiation markers (OPN, ALP, and COL-I); and promoted bone regeneration in OVX-induced osteoporotic mice.^164^ Further studies revealed that the osteogenic activity of YNVs was not dependent on saponin, a known bone-promoting active ingredient in yam, but was mediated by the BMP-2/p-p38-dependent Runx2 pathway.^164^

## The potential applications of EVs in osteoporosis

### Diagnostic tools

**Fig. 5:**
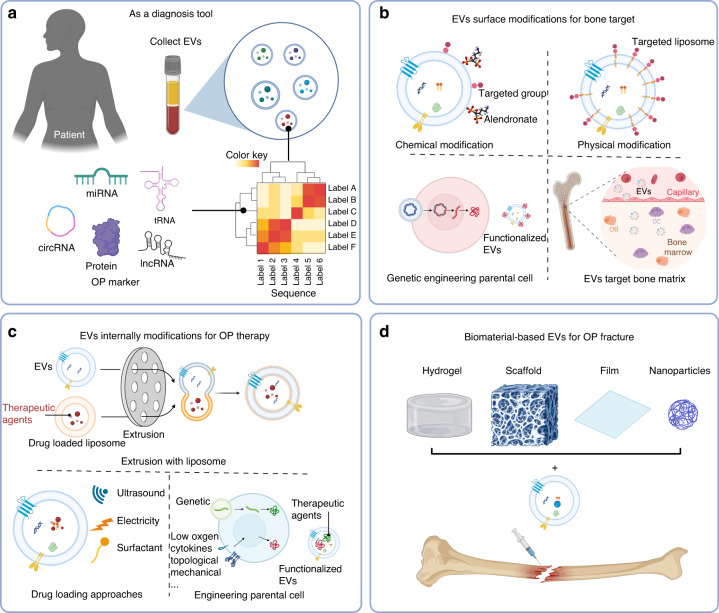
The applications of EVs in osteoporosis treatment. **a** The relevant markers of osteoporosis were identified by sequencing and analyzing the content of EVs in blood. **b** Through chemical modification, physical modification and genetic engineering of parental cells, bone-targeting ligands can be modified on the surface of EVs. **c** Osteoporosis therapy-related EVs were obtained by extrusion with drug-loaded liposomes, ultrasound, electrical stimulation to load drugs and genetic engineering of parental cells. **d** EVs can treat osteoporotic bone defects by being compounded with biomaterials, such as hydrogels, scaffolds and nanoparticles

Recent studies have demonstrated that the presence of EVs in body fluids (such as blood, urine, saliva, and ascites) facilitates the identification of biomarkers and therapeutic targets for various diseases.^168–171^ To date, EVs in blood samples have been used to identify diagnostic markers of osteoporosis (Fig. 5a). Cargo in EVs, such as proteins, miRNAs, circRNAs, and tRNAs, are commonly identified as biomarkers of osteoporosis. Previous studies have demonstrated that the level of miR-214 in the serum EVs of osteoporotic patients is significantly higher than that in healthy controls, and the level of miR-214 in these circulating EVs is a biomarker of bone loss.^94^ This study also confirmed that osteoclasts secreted miR-214 and could selectively regulate osteoblast function.^94^ Additionally, a large-scale clinical study of postmenopausal women with osteoporosis showed that serum exosomal miRNAs were differentially expressed in postmenopausal osteoporosis patients and confirmed that miR-3-766p and miR-3-1247p were related to bone mineral density and that miR-5-330p, miR-5-3124p, and miR-5-p could be used as candidate diagnostic biomarkers.^172^

Proteins in circulating EVs can also serve as important biological markers of osteoporosis. For example, proteomic sequencing of serum EVs from patients with osteoporosis revealed that 19 proteins were consistently upregulated in the osteopenia and osteoporosis groups compared with the healthy group.^173^ Further verification revealed that the average concentration of profilin 1 in the serum EVs of patients with osteoporosis was 96.22 pg/mL, which was significantly higher than that in the control group.^173^ In addition, the results of a multi-sample study (30 subjects with osteoporosis and ten subjects with osteopenia) showed that the serum EV proteins PSMB9, PCBP2, VSIR and AARS in patients with osteoporosis could be used to predict osteoporosis, which achieved an AUC of 0.805 in the classification of osteoporosis.^174^ Unfortunately, this study did not validate the expression of osteoporosis predictor proteins in EVs. An in vitro study revealed that the metabolites cytidine, isocytosine, thymine, succinate, and citrulline in EVs could be biomarkers of periodontal tissue destruction.^175^

Furthermore, other RNA components in EVs, such as circRNAs.^176,177^ and tRNAs,^178^ can be used as blood diagnostic biomarkers for osteoporosis. For example, Hua et al. analyzed circRNAs in the serum EVs of osteoporosis patients using a circRNA microarray and qRT‒PCR.^176^ Their results confirmed that Hsa_circ_0006859 expression was significantly upregulated in the exosomes of osteoporosis patients compared with healthy controls, suggesting that Hsa_circ_0006859 could serve as a biomarker for postmenopausal osteoporosis.^176^ In addition, in vitro experiments confirmed that hsa_circ_0006859 inhibited osteogenesis and promoted adipogenesis by upregulating ROCK1 by sponging miR-431-5p.^176^

### Therapeutic drugs and engineered optimization

#### Therapeutic drugs

Osteoporosis is thought to be caused by disruption of the balance between bone resorption and bone formation. Therefore, the current treatment involves inhibiting osteoclast activity and promoting osteoblast differentiation.^179^ The use of natural EVs derived from MSCs, osteoblasts, endothelial cells, muscle cells, and immune cells to treat osteoporosis is discussed in the third section. The main functional units of these EVs for osteoporosis treatment include RNAs, miRNAs and protein components. Natural EVs have multiple advantages as therapeutic drugs, such as good biocompatibility, stable physicochemical properties, prolonged blood circulation time, and low immunogenicity.^180,181^ However, EVs also have several obvious limitations, such as being more concentrated in the liver, spleen and kidney in vivo and lacking the ability to target bone tissue.^182^ Thus, an increasing number of engineering strategies are being used to modify EVs to effectively treat osteoporosis. These strategies can be divided into two categories: (1) surface modification of EVs to improve the targeting of bone tissue ; and (2) internal modification of EVs to improve their antiosteoporotic activity.

#### External engineering approaches

The surface modification of EVs for bone-targeted delivery has been well studied and includes chemical modification, physical modification, and genetic engineering (Fig. 5b).^14,183^ Among these methods, click chemical reactions are used mainly to graft bone tissue-targeting molecules on the surface of EVs to improve bone targeting.^184^ min et al. added azide to the surface of MSCs through metabolic glycoengineering.^184^ They fabricated EVs loaded with the smoothness agonist SAG by the extrusion method and then attached a bone-targeting ligand (alendronate, ALD) by copper-free click chemistry.^184^ These bone-targeted EVs (ALD-EM-SAG) exhibited excellent binding affinity to artificial and natural apatite substrates of bone tissue and could significantly alter the bone microenvironment and promote bone regeneration.^184^

Physical modification mainly involves noncovalent binding of bone-targeting functional groups via hydrophobic interactions (fusion with liposome membranes, lipid insertion), electrostatic interactions, and ligand‒receptor interactions.^14,183^ This approach is characterized by its simplicity and convenience, although it exhibits a lower level of stability than chemical modification. For example, lipid insertion involves the incubation of bone-targeted functionalized liposomes with EVs, resulting in the generation of bone-targeted EVs through hydrophobic interactions.^185,186^ Wang et al. used alendronate (ALN)-grafted pegylated phospholipids (DSPE-PEG-ALN) to bind EVs derived from platelet lysates and obtain bone tissue-targeted PL-exo-ALN.^186^ The HA-binding affinity of the PL-exos in vitro and their ability to undergo bone-targeted accumulation in vivo were significantly enhanced by the ALN modification.^186^ Furthermore, the enrichment of growth factors in PL-exo-ALN could effectively promote the osteogenic differentiation of BMSCs and angiogenesis of EPCs.^186^

In genetic engineering, bone tissue-targeted ligands are displayed on the EV source cell membrane through a plasmid vector. BMSCs in the bone marrow highly express SDF1, which can recruit CXCR4 to peripheral HSCs for homing and promote bone metastasis in several CXCR4-positive tumor cells.^187^ Considering the critical role of the CXCR4-SDF1 axis in chemotactic behavior, CXCR4-positive EVs were developed for bone tissue disease therapy.^156,188,189^ Su et al. genetically fused hCXCR4 to the protein ClyA, which is a BEV surface protein, to generate ClyA-hCXCR4 and subsequently constructed pET28a-ClyA-hCXCR4 (pClyA-hCXCR4).^156^ CXCR4-positive EVs were subsequently generated from the transgenic strain ECN-pClyA-hCXCR2.^156^ In addition, SOST siRNA was electroporated into BEV-hCXCR4 cells to obtain BEV-hCXCR4-SOST siRNA (BEV-CSs), which regulated the WNT signaling pathway to induce osteogenic differentiation in BMSCs.^156^ It was found that customized BEV-CSs exhibited strong bone-targeting abilities, could be internalized by BMSCs, promoted osteogenic differentiation, and successfully reversed osteoporosis in a mouse model.^156^

#### Internal engineering approaches

Internal engineering of EVs mainly includes the physical loading of drugs and the use of genetic engineering or biophysical stimulation to modify EV cargoes, including proteins and miRNAs (Fig. 5c). The methods for loading EVs with drugs include incubating drugs with donor cells,^190^ fusing drug-loaded liposomes with donor cells or EVs,^191,192^ physical extrusion,^193,194^ ultrasonic treatment,^195^ electroporation,^185,196^ or surfactant treatment.^197^ Su et al. used physical extrusion to develop bone-targeted EVs and loaded one of the Wnt agonizts into these EVs.^193^ BMSCs internalization of the engineered EVs promoted osteogenic differentiation and inhibited adipogenic differentiation, which could effectively alleviate the impairment of osteoblastic bone formation and bone loss in the context of inflammatory bowel disease.^193^

Genetic engineering involves integrating the target gene into the donor cell of EVs to improve their activity.^198,199^ Xie et al. integrated the bone formation-stimulating protein neural EGFL-like 1 (NELL1) and the BMP2 protein into BMSCs and collected the secreted EVs.^199^ The authors found that these NELL1-modified EVs could significantly increase the osteogenic abilities of BMSCs by activating the miR-25-5p-SMAD2 signaling axis.^199^

Furthermore, several biochemical or biophysical methods, including hypoxic preconditioning,^200^ cytokine pretreatment,^201^ biomaterial topography^202^ and mechanical stimulation,^124,203^ have been used to modify EVs for the treatment of osteoporosis. Examples include the use of mechanical stimulation to increase the activity of EVs and promote osteogenesis.^124^ Studies have shown that MS-BMDM-EXOs more robustly increased the osteogenic potential of BMSCs after mechanical stimulation than those in the non-mechanical stimulation group.^124^ Proteomic analysis revealed that mechanical stimulation increased the enrichment of ubiquitin carboxy-terminal hydrolase isozyme L3 (UCHL3) in EVs and that UCHL3 could regulate BMSC osteogenic differentiation through SMAD1 signaling.^124^

In general, external and internal modification of EVs enhance their biological activity and the targeting of bone tissue. Therefore, multiple engineering methods are often combined to maximize therapeutic potential.

### Biomaterial-based EVs for osteoporotic fracture

In addition to direct injection, EVs can also be loaded on hydrogels,^204–206^ scaffolds,^198,207,208^ films,^209,210^ or other biomaterials for bone repair (Fig. 5d). Biomaterial-assisted EVs as therapeutic vehicles for bone regeneration have been well characterized, and here, we provide only a brief review.^211^ These biomaterial scaffolds overcome the shortcomings of native EVs by prolonging EV storage time and modifying the release characteristics, enabling EVs with desirable drug acceptability. Hydrogel is a nonimmunogenic natural polymer that has excellent tissue- and cytocompatibility. Xie et al. developed GelMA and HAMA-based hydrogels to deliver nanohydroxyapatite and urine-derived stem cell-derived EVs for bone repair.^212^ The hydrogel exhibited delayed EV release in vitro, with sustained release for up to 17 days.^212^ Furthermore, the EV-loaded hydrogel promoted the osteogenic differentiation of BMSCs in vitro and the regeneration of defective calvaria in vivo.^212^

## Conclusion and future perspectives

Osteoporosis is a bone disease characterized by decreased bone density and mass, leading to brittle bones and an increased risk of fractures. As important intercellular communication factors, EVs are essential for determining the etiology, diagnosis, and treatment of osteoporosis. Studies in the past decade have shown that EVs derived from different sources play different roles in osteoporosis. This article reviewed the roles of EVs derived from various tissues or other organisms in osteoporosis and outlined methods for diagnosing and treating osteoporosis by using EVs.

Studies on the role of EVs in osteoporosis have focused mainly on the abundant contents of EVs, which play crucial roles in regulating both bone formation and resorption. For instance, EVs derived from various cell types, such as osteoclasts, osteoblasts, MSCs, M0 and M2 macrophages, endothelial cells, and smooth muscle cells, carry miRNAs, proteins, and Linc-RNAs. These components effectively induce osteoblast differentiation while inhibiting osteoclast differentiation to promote bone formation. However, exosomes derived from osteoclasts, osteoblasts, cancer cells and M1 macrophages exert contrasting effects by inducing osteoclast differentiation while inhibiting osteogenic differentiation to facilitate bone resorption. (Fig. 4). In addition, EVs can regulate the inflammatory response and immune function and have specific impacts on the development of osteoporosis. For example, apoptotic EVs derived from BMSCs inhibited the formation of adjacent osteoclasts by inhibiting proinflammatory macrophage polarization and TNF-α secretion via the AMPK/SIRT1/NF-κB pathway.^213^ Studies have also confirmed that macrophage-derived EVs have immunomodulatory effects and can regulate the balance of regulatory T cells (Tregs) and helper T cells (Th17 cells) in the bone microenvironment to suppress bone loss in osteoporosis.^214^ However, due to the diversity of EV sources and lack of a standardized approach for EV isolation, further research is needed to determine the specific role and application value of EVs in osteoporosis.

Although the mechanism by which EVs affect osteoporosis has not been fully elucidated, there is a growing body of research focused on leveraging EVs to diagnose and treat this condition. The diagnosis of diseases based on EVs begins with the classification of tumor malignancy.^215^ Therefore, research and technology related to the use of EVs in disease diagnosis are relatively sufficient. Recently, EVs have been used as biomarkers for the early diagnosis and monitoring of osteoporosis. The bioactive molecules, miRNAs, proteins, and Linc-RNAs that are enriched in EVs are closely related to bone metabolism. Therefore, by detecting EVs in body fluids, the risk or progression of osteoporosis can be detected early, and individualized treatment can be carried out. Interestingly, EVs may also be tools for the precise determination of different types of osteoporosis. Postmenopausal osteoporosis is mainly caused by reduced ovarian production of estrogens, and bone loss is most prominent in trabecular bone.^216^ Disuse osteoporosis is mainly caused by enhanced bone resorption and the inhibition of bone formation after the reduction of bone mechanical force, and the mechanism is different and independent of the mechanism that leads to postmenopausal osteoporosis.^217^ One study showed that EVs derived from the blood of mice subjected to hindlimb tail suspension uniquely expressed CXCL1, lipocalin 2, and MMP-3, whereas ovariectomized mouse-derived circulating EVs were only enriched in P-selectin.^218^ To date, EV-mediated diagnosis of osteoporosis has primarily focused on blood samples, and there have been limited reports on other tissues. Moreover, the analysis of osteoporosis markers in EVs relies heavily on multiomics approaches, resulting in increased diagnostic costs for osteoporosis assessment.

Furthermore, EVs have been extensively studied for osteoporosis treatment. As mentioned previously, EVs derived from various cell sources show excellent abilities to promote bone formation and inhibit bone resorption. EVs derived from MSCs have been the most commonly reported for the treatment of osteoporosis. MSC-derived EVs compensate for the shortcomings of the direct use of MSCs for osteoporosis treatment, such as limited cell viability, immune rejection, and phenotypic uncertainty after transplantation.^219^ However, these naturally derived EVs have limitations in osteoporosis treatment, such as a lack of bone targeting and effective therapeutic activity, which results in insufficient therapeutic efficacy. Therefore, biomimetic synthesis and optimization of EVs are currently effective means to improve the therapeutic activity and bone tissue targeting. To improve the bone-targeting ability of EVs in vivo, researchers have developed many engineering strategies, such as surface modification via chemical, physical, and genetic methods. To enhance bioactivity, many approaches, such as extrusion with drug-loaded liposomes, ultrasound, electrical stimulation to load drugs, and miRNA or protein overexpression by genetically engineering the parental cells, have been used. However, these engineering modification strategies may also have drawbacks, such as uncertain immune responses and high production costs. Surface-engineered modifications of EVs may cause the immune system to recognize them as foreign bodies, triggering a host immune response that can lead to clearance or reduced efficacy. These engineering modifications may cause toxicity or adverse reactions to EVs, posing potential risks to the host. In addition, engineering modifications require additional time, expense and technology, which may increase production costs. Therefore, when engineering EVs, safety, immunogenicity, stability and production cost must be considered, and their application prospects should be evaluated through strict experimental and clinical studies.

Finally, although the potential use of EVs in osteoporosis management is promising, several challenges still need to be addressed. Methods for EV preparation and purification have not yet been fully developed, and it is essential to consider how their source and preparation process may impact their biological activity and stability. Furthermore, understanding the function and regulatory mechanism of the bioactive substances within EVs is necessary to determine the mechanism by which they can treat osteoporosis. Therefore, future research should be devoted to exploring more efficient and stable preparation methods for EVs, conducting in-depth studies of their biological mechanism, and undertaking clinical trials to facilitate the use of EVs in osteoporosis treatment.
